# Deep learning on reflectance confocal microscopy improves Raman spectral diagnosis of basal cell carcinoma

**DOI:** 10.1117/1.JBO.27.6.065004

**Published:** 2022-06-30

**Authors:** Mengkun Chen, Xu Feng, Matthew C. Fox, Jason S. Reichenberg, Fabiana C. P. S. Lopes, Katherine R. Sebastian, Mia K. Markey, James W. Tunnell

**Affiliations:** a The University of Texas at Austin, Department of Biomedical Engineering, Austin, Texas, United States; b The University of Texas at Austin, Division of Dermatology, Dell Medical School, Austin, Texas, United States; c The University of Texas MD Anderson Cancer Center, Department of Imaging Physics, Houston, Texas, United States

**Keywords:** Raman spectroscopy, reflectance confocal microscopy, deep learning, basal cell carcinoma

## Abstract

**Significance:**

Raman spectroscopy (RS) provides an automated approach for assisting Mohs micrographic surgery for skin cancer diagnosis; however, the specificity of RS is limited by the high spectral similarity between tumors and normal tissues structures. Reflectance confocal microscopy (RCM) provides morphological and cytological details by which many features of epidermis and hair follicles can be readily identified. Combining RS with deep-learning-aided RCM has the potential to improve the diagnostic accuracy of RS in an automated fashion, without requiring additional input from the clinician.

**Aim:**

The aim of this study is to improve the specificity of RS for detecting basal cell carcinoma (BCC) using an artificial neural network trained on RCM images to identify false positive normal skin structures (hair follicles and epidermis).

**Approach:**

Our approach was to build a two-step classification model. In the first step, a Raman biophysical model that was used in prior work classified BCC tumors from normal tissue structures with high sensitivity. In the second step, 191 RCM images were collected from the same site as the Raman data and served as inputs for two ResNet50 networks. The networks selected the hair structure and epidermis images, respectively, within all images corresponding to the positive predictions of the Raman biophysical model with high specificity. The specificity of the BCC biophysical model was improved by moving the Raman spectra corresponding to these selected images from false positive to true negative.

**Results:**

Deep-learning trained on RCM images removed 52% of false positive predictions from the Raman biophysical model result while maintaining a sensitivity of 100%. The specificity was improved from 84.2% using Raman spectra alone to 92.4% by integrating Raman spectra with RCM images.

**Conclusions:**

Combining RS with deep-learning-aided RCM imaging is a promising tool for guiding tumor resection surgery.

## 1 Introduction

Mohs micrographic surgery (MMS) is the most effective method to treat nonmelanoma skin cancer.^1^^,^^2^ With Mohs, the physician removes tumor tissue in stages. Within each stage, a layer of skin is excised and processed using frozen section histopathology, and the physician keeps removing layers until no tumor tissue is identified at the surgical margin. Although Mohs has a cure rate >98%,^2^ the histopathological analysis for each stage is relatively time-intensive (30 to 60 min), requiring expensive clinical infrastructure and physician training. Optimization of care delivery using novel technology would add benefit for patients with high-risk skin cancer.

Raman spectroscopy (RS) is a promising tool for skin cancer diagnosis due to its high molecular specificity, low invasiveness, and low sample preparation requirement.^3^ RS is a sensing technique that uses light scattering to determine the molecular vibrational modes to provide chemical fingerprints.^4^ Prior studies have shown promising performance in differentiating basal cell carcinoma (BCC) from surrounding normal tissues based on both analyzing the difference of BCC Raman spectra from normal tissue Raman spectra and using the nature and biochemical processes responsible for the spectral differences. Several recent studies have reported diagnostic sensitivities near 100% with specificities above 90%.^5^^–^^8^ Because Mohs is very successful with a 98% cure rate, to be adopted as a low-infrastructure alternative to Mohs, RS should also be highly accurate. Currently, RS is limited by its specificity. False positive Raman spectra in RS arise most often from tissues with high nuclear density such as the epidermal layer of skin, hair structures, and areas of inflammation.^9^^,^^10^

Reflectance confocal microscopy (RCM) is a complementary optical imaging technique that offers noninvasive visualization of skin structures *in vivo* at subcellular resolution,^11^ and it has been advanced into clincial practice for noninvasive diagnosis of skin cancer.^12^ RCM was first reported to be used on BCC by González and Tannous,^13^ and a meta-study reported the sensitivity of 92% (range 87% to 95%) and specificity of 91% (range 84% to 96%) for BCC diagnosis by human using RCM images.^14^ Automatic segmentation on RCM images of different structures was explored by D’Alonzo et al.,^15^ and they achieved a 0.97 area under the curve (AUC) for reciever operator characteristic (ROC) analysis for classification. Automatic diagnosis of BCC by RCM images was also studied by Campanella et al., and they achieved an AUC of around 0.89 ROC.^16^

Although systems using the combination of RS and RCM have been developed,^17^^,^^18^ the utilization of this combination has not been studied for the automated diagnosis of BCC. We proposed a method to combine RS and RCM sequentially to improve the diagnostic accuracy of BCC diagnosis. The RS model selected BCC spectra with high sensitivity, and the RCM model selected non-BCC structures from all positive predictions to improve the specificity. Our previous report using RS alone^19^ discriminated BCC from normal tissues with 100%/84% sensitivity/specificity (30 patients and 223 spectra).^20^ Many of the false positives originated from hair structures, epidermis, and inflammation. Further examination of the RCM images that were acquired at the same site as the RS data showed that hair structures and epidermis were often easily discernable in the structural RCM images. Hair exhibited circular structures from the shaft cross section, and the epidermal layers exhibited ribbon patterns from the highly scattering stratum corneum. Both exhibited honeycomb shaped cellular patterns. We are not aware of prior studies directed at recognizing the hair and epidermal structures in RCM images as a method to improve the diagnosis of another technique. Therefore, we trained two convolutional neural network (CNN) models to identify hair structures and epidermis, respectively. After training, we collected all of the RCM images classified as positive according RS (including all true positive and all false positive) and used the two CNN models to identify the hair structures and epidermis images. Then, we moved the corresponding sites from the positive category to the negative category. By doing so, we converted more than 52% of the false positves to true negatives, thus increasing specificity to 92.4% (an 8% increase) while keeping the sensitivity at 100%.

## 2 Methods

### 2.1 Clinical Data Set

**Fig. 1:**
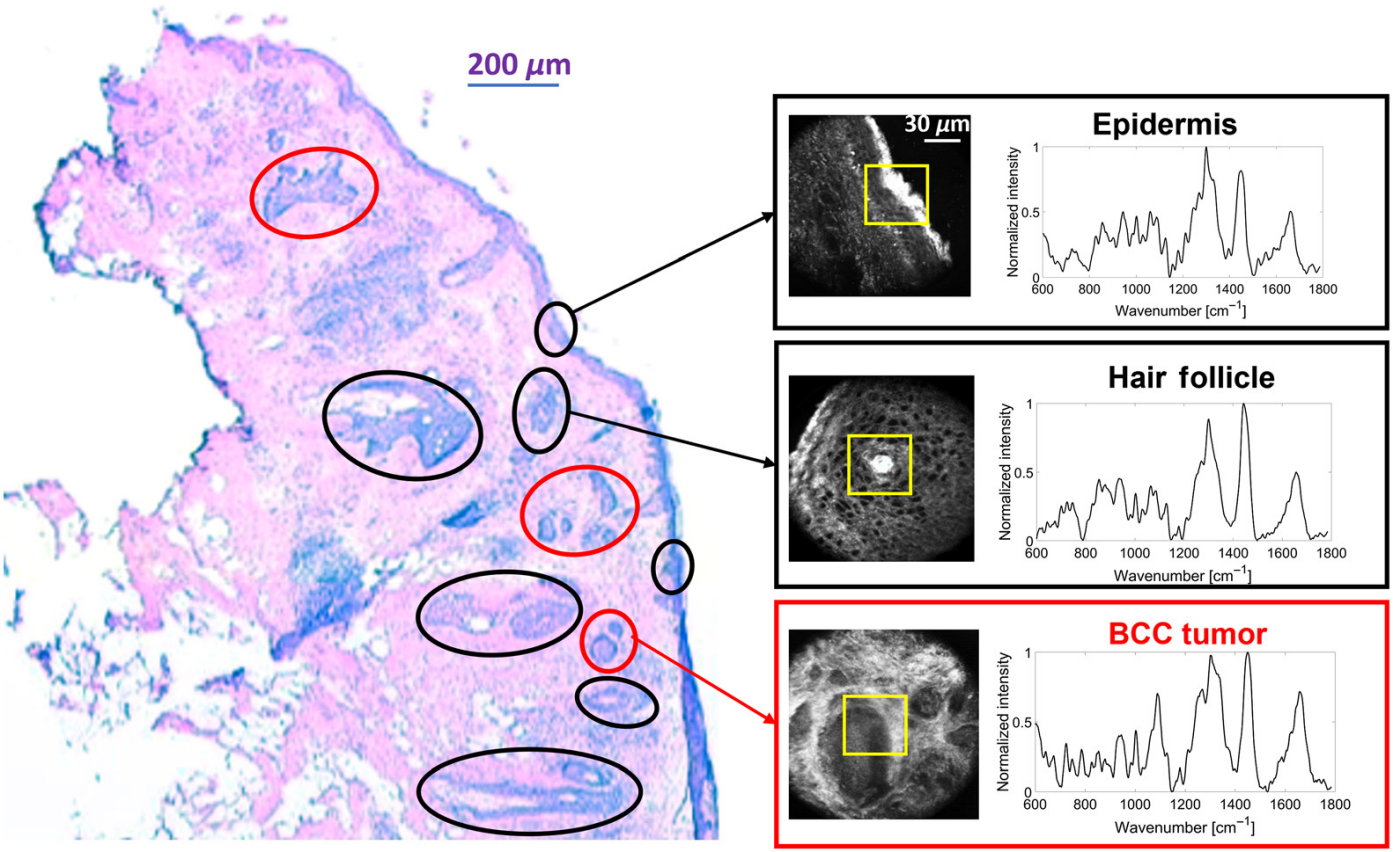
Illustration of prior experiment (Feng et al.’s 2019 study) where Raman spectra and RCM images were acquired from Mohs micrographic sections. The red circle is BCC tumor. The black circle is normal tissue structures including hair structure and epidermis. The yellow square is regions that were assessed by Raman scanning.

For this analysis, we combined RS and RCM images from two prior studies (Feng et al.^20^^,^^21^) consisting of 292 site-matched RS and RCM images (141 and 151 for the two studies, respectively) from Mohs surgical sections. Figure 1 shows a typical experiment whereby an RS spectrum and RCM image were acquired from the exact same location over various skin constituents including hair structures, dermis, epidermis, fat, inflammation, and BCC. Raman spectra and RCM images were collected using a custom-built Raman confocal microscope with a 830-nm excitation wavelength.^19^ The lateral, axial, and spectral resolutions of the system were ∼1  μm, 8  μm, and $8\;{\mathrm{cm}}^{-1}$, respectively. The laser power was approximately 45 mW at the source. Raman spectra were acquired by scanning a field of view from $60\times 60\;\mu m^{2}$ to $100\times 100\;\mu m^{2}$ (2-μm steps, 2 s per step). Raw Raman spectra underwent wavenumber calibration, dark noise removal, cosmic ray removal, smoothing, and fluorescence background removal. The effective spectral range was 800 to $1790\;{\mathrm{cm}}^{-1}$. Data were normalized to the AUC. After each scan, 900 to 2500 Raman spectra were analyzed within one scan area (30×30 to 50×50 spectra). Raman pseudo-color images were generated by k-means clustering with the number of clusters being determined by visual comparison of the pseudo-color image and histopathology. Each cluster was represented by the centroid Raman spectra and assigned a different color. The spectra used to build the biophysical model were the centroid Raman spectra from corresponding tissue structures. The spectra shown in Fig. 1 are the centroid Raman spectra.

The Feng et al.’s 2019 study reported Raman imaging on 30 frozen tissue blocks from 30 patients. A previously developed biophysical model^19^ was used on Raman spectra to discriminate BCC and normal tissues, and it achieved 100% sensitivity and 84% specificity when prioritizing sensitivity. RCM imaging and frozen section hematoxylin and eosin staining were also acquired for reference, but they were not used for diagnosis for that study.

**Fig. 2:**
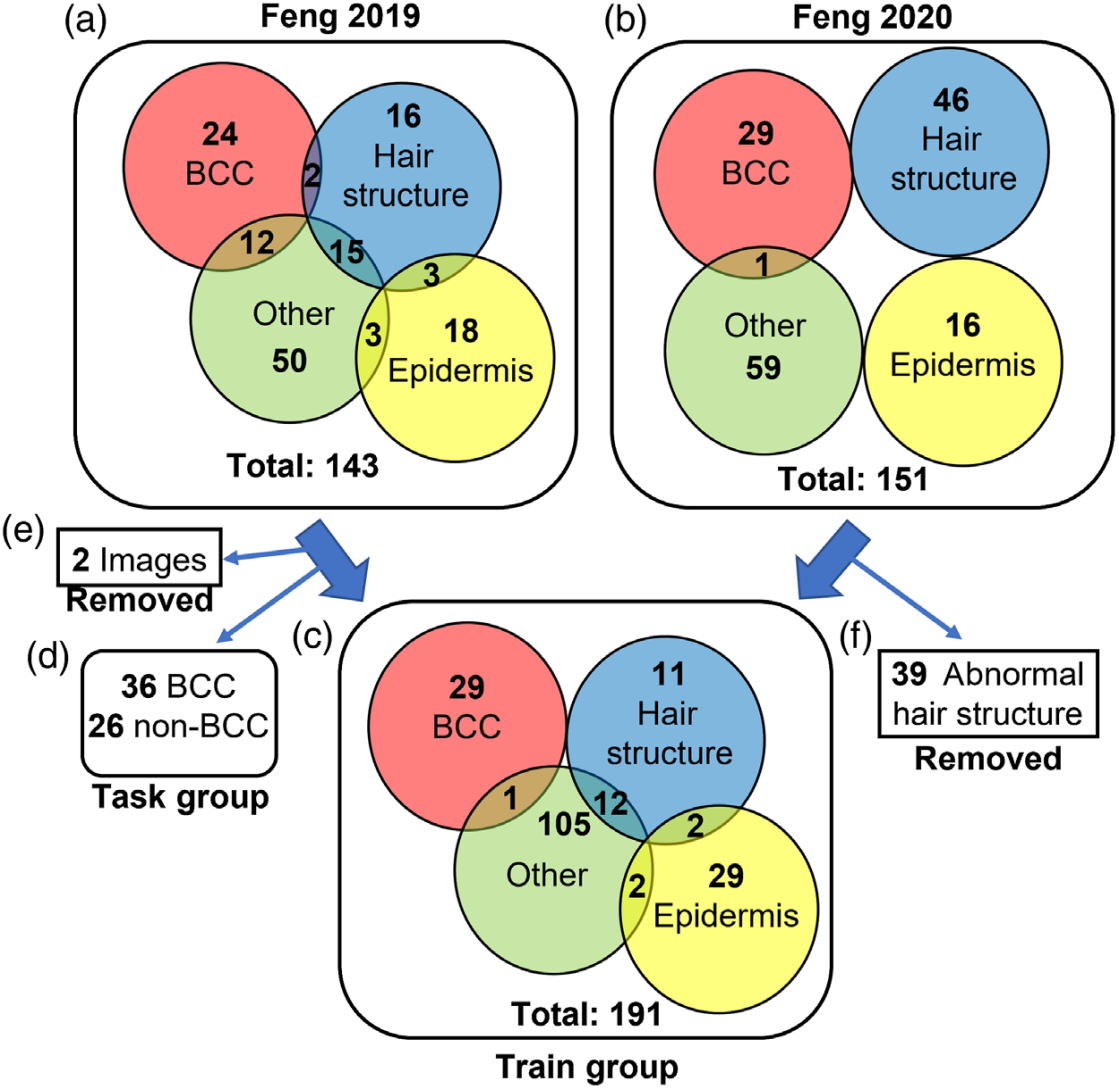
Description of the RCM image sets used for the train and task groups. Images in box (a) were from Feng et al.’s 2019 study. Images in box (b) were from Feng et al.’s 2020 study. Images in box (c) were the train group used for our models. Images in box (d) were the task group. Images in box (e) were removed because these two images contained both hair structure and BCC. Images in box (f) were removed for having abnormal hair structures. The intersections of the colored circles indicate that more than one structure is present in one image.

Figure 2 shows the RCM images used for this study. The RCM images in the “task group” correspond to the Raman spectra that were predicted as containing BCC, including both false positives and true positives, when prioritizing sensitivity. The rest of the RCM images are in the “train group.” To enlarge the train group, we also used the RCM images from Feng et al.’s 2020 study.^21^ These images were obtained using the same system and in the same scale as Feng et al.’s 2019 study. We first removed two images from Feng et al.’s 2019 dataset because they contained both BCC and a hair follicle structure, and our current model requires that each image only contains one of these classifications. Overall, there were 230 RCM images (30 BCC and 200 non-BCC) in the train group and 62 RCM images (36 BCC and 26 non-BCC) in the task group. There was no overlap between the images in the train group and the task group.

Furthermore, we removed 39 images of hair structures (including hair follicle, hair shaft, and hair core) from the train group that had abnormal patterns and looked very different from the other hair structure images, which resulted in 191 RCM images in the final train group. There were three reasons why an image was labeled abnormal. First, the image frame failed to cover the complete circular hair structure (e.g., only the outer arc was visible in the image). Second, large areas of pixels were missing because of problems in the acquisition process. Third, some hair structures had an abnormal growth appearance that did not conform to the regular circular shape (Fig. S1 in the Supplemental Material presents all images that were noted as abnormal and removed from the train set). Attempts to train the model with these 39 images included resulted in nonconverging training loss and overfitting, which appeared after just the first few epochs.

#### 2.2 Data Preprocessing

To prepare images for use in training the CNN, we performed several preprocessing operations on the images in the train group. First, we randomly divided the images in the train group into the training set, validation set, and test set. For the hair model, the image numbers ratio of train/validation/test was 121/35/35. For the epidermis model, the ratio was 139/26/26. These ratios provided the fastest convergence of the training loss. We trained two binary classification models. One model is trained to classify “hair structure” versus “non-hair structure”; the other one is trained to classify “epidermis” versus “non-epidermis.” However, both categories are unbalanced in the training set (23 hair structure images and 168 non-hair structure images, 33 epidermis images and 158 non-epidermis images). Therefore, in the training set only, we performed data augmentation on both categories for the two models, respectively (when training the hair structure model, we only augmented hair structure images, and when training the epidermis model, we only augmented epidermis images) by flipping, rotating, scaling, and shifting. After positive category augmentation, we resized all images to 224×224×3 (height × width × channel) and performed routine normalization and standardization (each pixel minus mean value and divided by standard deviation). Although these were grayscale images, we kept three RGB channels to match the input size for transfer learning. The grayscale image was repeated three times in each channel. We performed 10 times bootstrapping to demonstrate that there was no bias when splitting train/validation/test sets (results shown in Fig. S3 in the Supplemental Material).

#### 2.3 Model Architecture and Training

Given our relatively small training set, transfer learning is a suitable choice for our models. Transfer learning uses the weights of a pre-trained network to transfer into our own models, and training on our data added only a small number of layers with a small number of parameters,^22^ which has been demonstrated as a powerful tool in the medical imaging field.^23^^–^^26^ We used ResNet50^27^ as the model architecture. We removed the top fully connected (FC) layers and added one FC layer with two neurons as the output with the SoftMax activation function and the cross-entropy loss. The ResNet50 architecture was used for feature extraction, and the FC layer was added for forming the final output as a probability using the extracted features.

To train the hair structure model, we imported all weights for the 50 layers trained from ImageNet and left the FC layer trainable. To train the epidermis model, we imported all weights for the first 40 layers trained from ImageNet and left the rest (10 layers) and the FC layer trainable. For both models, we used mini-batch Stochastic gradient descent to minimize the cross-entropy loss and used momentum with β=0.9 as the optimizer. In addition, we added L2 loss with λ equal to 0.01 for regularization. When inputting images, we randomly selected a batch of 16 images and performed augmentations by flipping, rotating, shifting, and scaling. All of the augmentations were randomly selected and were applied on random images in the batch. This function was fulfilled by TensorFlow ImageDataGenerator.^28^ This augmentation was used for increasing the variation of the whole dataset, while the augmentation in 2.2 was used on the positive images to make the dataset balanced. The trainings were performed with one NVIDIA Tesla K80 Graphic Card provided on Google Cloud Platform. Each model was trained for 0.5 h with 100 epochs. We chose these hyper-parameters because they helped the training loss converge faster than other hyper-parameters.

The final diagnosis was made based on the union of the two models. Images within the task group classified as either hair structure or epidermis were assigned as non-BCC; then we converted their corresponding Raman spectra from BCC to non-BCC.

## 3 Results

### 3.1 Training Process

**Fig. 3:**
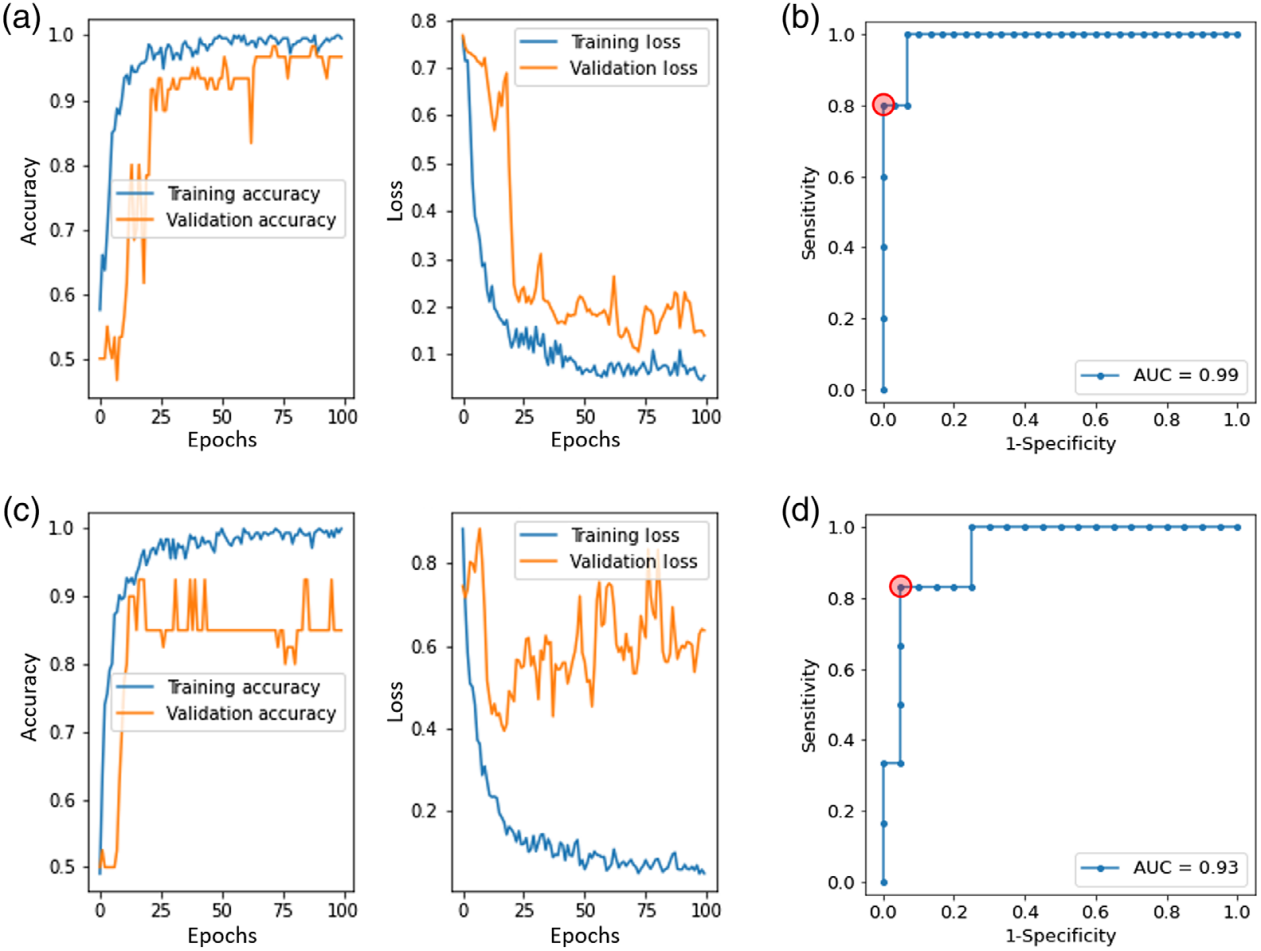
Training process details. (a) Training and validation loss and accuracy for the hair structure model. (b) ROC curve for the hair structure model on the test set. (c) Training and validation loss and accuracy for the epidermis model. (d) ROC curve for the epidermis model on the test set. The red circles in (b) and (d) are the operating points corresponding to the thresholds selected for use in subsequent model validation on the task group data.

The model training process is shown in Fig. 3. Hair structures are more complex structures than epidermis, so training the model to identify hair structures required more epochs to converge than the epidermis model did, with its loss starting to increase after about 25 epochs because of overfitting. The ROC curves on test sets showed good performance of the models (AUC of 0.99 for the hair structure model and 0.93 for the epidermis model). We only used positive sample augmentation on the training and validation sets, so the number of positive and negative samples was still unbalanced in the test set, and the total sample number was small. For example, the test set for the hair structure model had only five hair structure (positive) samples and 30 non-hair structure (negative) samples. Thus, the number of operating points on the ROC curves for the test sets was limited (Fig. 3).

### 3.2 Model Performance

**Fig. 4:**
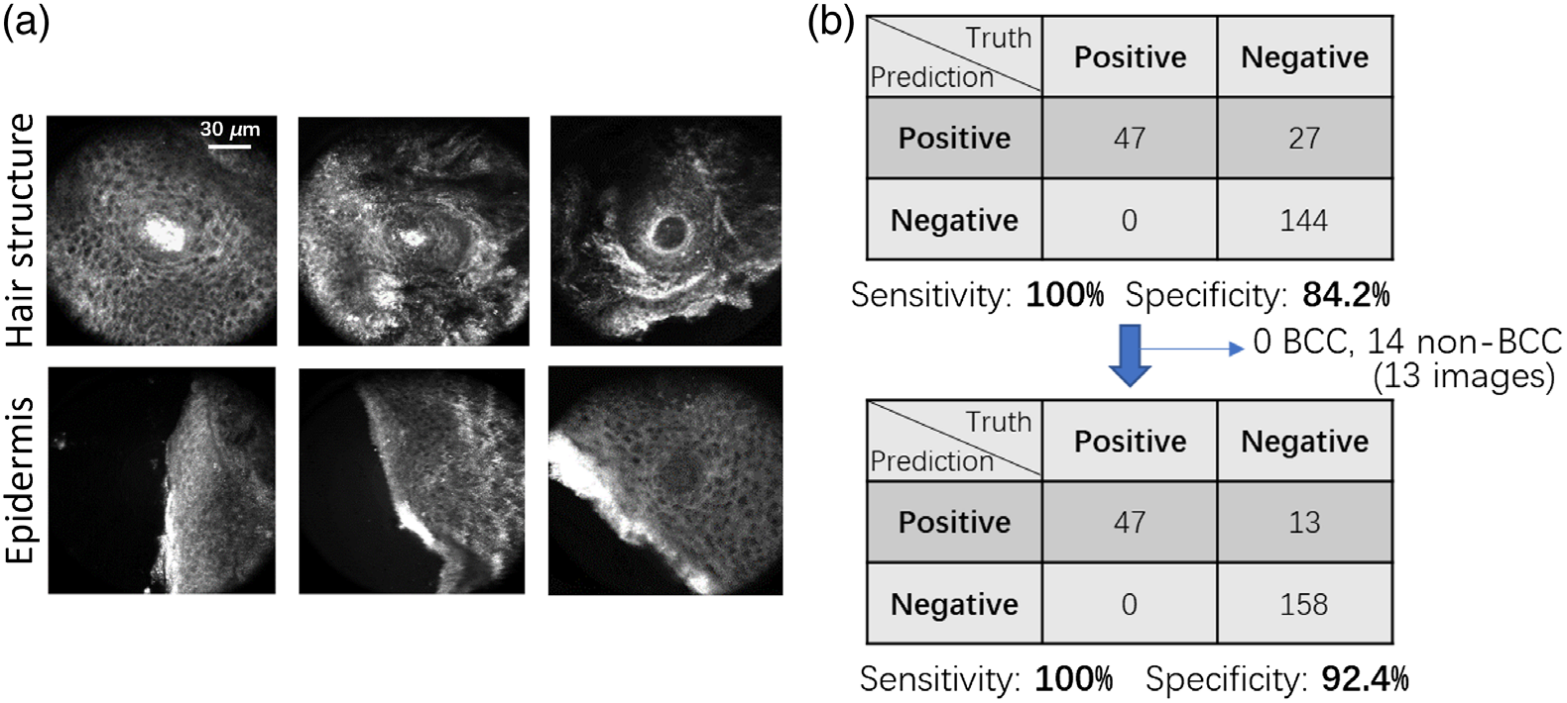
(a) Illustration of the RCM images within the task group that were identified as epidermis by the epidermis model and as hair structures by the hair structure model. (b) The confusion matrix for the Raman analysis changes after applying the models to identify epidermis and hair structures. The numbers in the confusion matrix are the numbers of spectra.

We applied both models on the independent task group. The thresholds for each model were selected based on the ROC curves on the test set in Fig. 3. We chose thresholds to achieve high specificity and acceptable sensitivity. For the hair structures model, we selected the threshold that provided sensitivity/specificity of 80%/100% on the test set, and for the epidermis model we selected the threshold that provided sensitivity/specificity of 95%/84% on the test set. The hair structure model predicted 8 non-BCC images and 0 BCC images as hair structures for the task group. The epidermis model predicted 5 non-BCC images and 0 BCC images as epidermis for the task group. Overall, there were 13 non-BCC images chosen by the models as non-BCC and 0 images identified as BCC for the task group. Figure 4(a) illustrates exemplar images from the task group that the models identified as containing hair structures or epidermis. The circular and ribbon structures are obvious in these images. Of the 27 false positive Raman spectra (associated with 26 RCM images) from the Raman model [Fig. 4(b)], the hair structure and epidermis models identified 13 images (associated with 14 Raman spectra) to be either hair structures or epidermis. Therefore, 14 Raman spectra were converted from false positives using Raman alone to true negatives by considering RCM. This reduced the false positives by 52% while keeping the sensitivity at 100% [Fig. 4(b)]. Within the 13 remaining unselected images, four of them are from structures other than epidermis/hair, six of them are abnormal hair structures, and three of them are abnormal epidermis structures. The unselected images are shown in Fig. S2 in the Supplemental Material.

## 4 Discussion

This study demonstrates the feasibility of combining Raman spectra and RCM images for skin cancer diagnosis. By training two CNN models with transfer learning to recognize hair structures and epidermis on RCM images, we removed 52% of the false positives from Raman while maintaining 100% sensitivity. The RCM models can accurately identify images containing circular patterns of hair structures and ribbon patterns of epidermis.

When preprocessing the training data, we removed hair structure images depicting less common shapes. We tested the hair model on the 39 removed hair structure images; only five images were selected by the model, which indicates that the removed images do not share the same pattern as the images in the training group. As mentioned in Sec. 2.1, we also trained the model without removing these images. The loss did not adequately converge, and the model classified five hair images and eight BCC images as non-BCC, resulting in reduced model performance. Although removing the 39 images helped the model to identify the typical circular shapes of most hair structures on RCM images, hair structures that have less common shapes could not be identified by the model. This can be improved in future studies by expanding the dataset to include a larger number of examples, especially of images depicting less common representations of hair structures under RCM images.

In this study we trained separate models to identify hair and epidermis. When training a single model to select both hair structure and epidermis, we observed reduced performance (decreased specificity in identifying hair and epidermis) as compared with separating the two models. We attribute this to the different appearances of these structures under RCM, requiring the single model to select both circular and ribbon shapes.

We used RCM images to identify hair structures and epidermis, not for BCC diagnosis. BCC under RCM does not have a unique and recognizable structure, making BCC difficult to be identified in RCM images even by someone experienced in reading RCM images.^29^^–^^32^ On the other hand, hair and epidermis have unique and recognizable structures under RCM that enable training a model to identify them. Furthermore, the Raman model already obtained 100% sensitivity; thus, an additional model that identified BCC would have been redundant. Therefore, RCM provided a complimentary model that identified common normal structures that Raman mistakes for BCC (i.e., epidermis and hair follicles). Thus, the combination of the two models improved the overall accuracy of identifying BCC. Future studies should focus on expanding this work to include more patients and the diverse representation of normal skin structures in RCM images that could further enhance the accuracy of training models for automated diagnosis of skin cancer.
