# Supplementary material for: Deep learning on reflectance confocal microscopy improves Raman spectral diagnosis of basal cell carcinoma
Source: J Biomed Opt. 2022 Jun 30;27(6):065004. doi: 10.1117/1.JBO.27.6.065004 (PMC9243521; doi:10.1117/1.JBO.27.6.065004)
Supplement: Supplementary file 1 [file JBO_027_065004_SD001.pdf]

Supplement A

Fig. S1 shows all the abnormal hair structure images we removed from our training group.

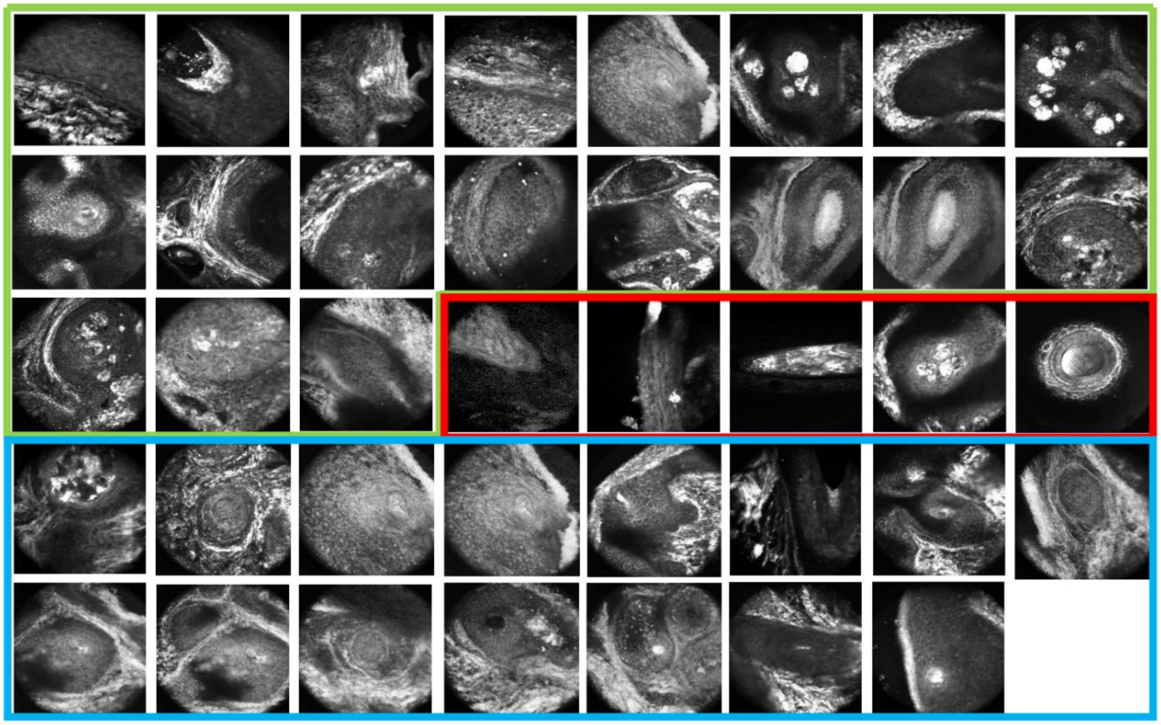

Fig. S1 Abnormal hair structure images we removed from our training group. Images in the green box were removed for reason 1: they failed to cover the complete circular hair structure. Images in the red box were removed for reason 2: large areas of pixels were missing because of the problems in the acquisition process. Images in the blue box were removed for reason 3: some of the hair structures were abnormally grown on the tissues.

Fig. S2 shows all the false positive images and those that were selected by the RCM models. In the red box are the images selected by the RCM models and in the blue box are those that were not selected. The figure demonstrates we have selected almost all images with ribbon structure (epidermis) and circular structure (hair).

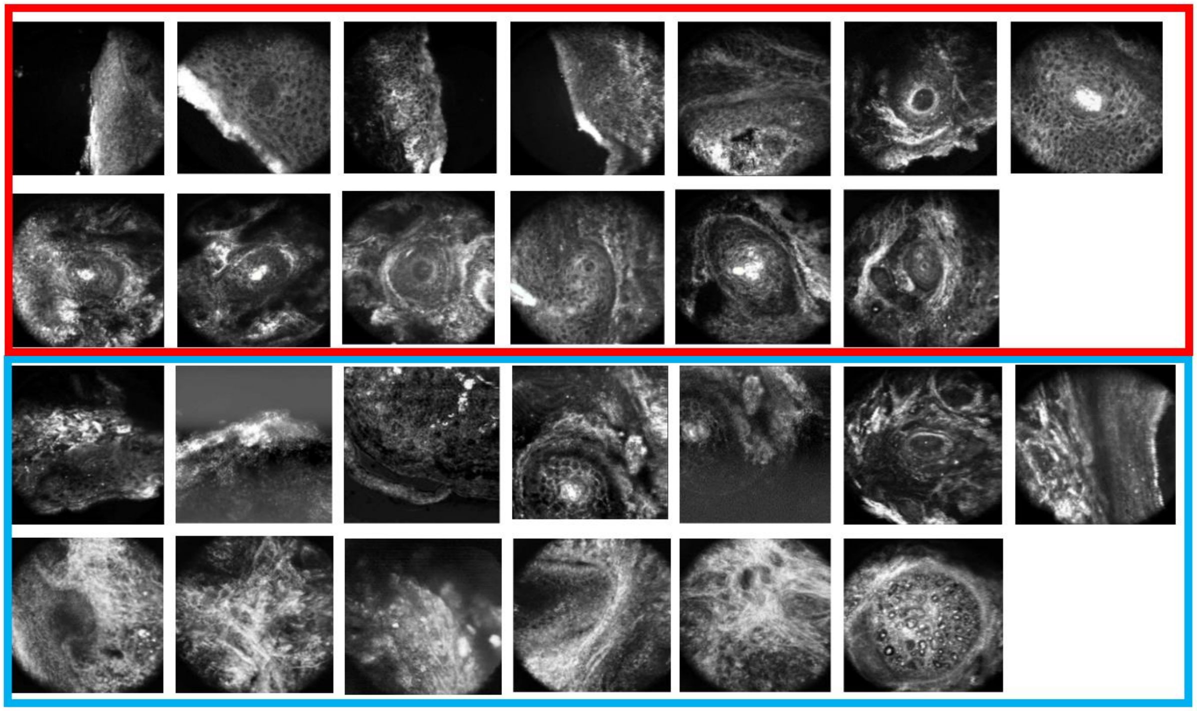

Fig. S2 All the false positive images. Images in the red box were selected by the models and removed form false positives. Images in the blue box were not selected by the model.

Supplement B

To investigate whether there was bias in the RCM models that was introduced when splitting train/validation/test set, we performed an additional 9 times bootstrapping for both hair structure and epidermis models. We randomly shuffled the dataset and repeated the methods reported in the paper for 9 additional times. Including the results reported in the paper, we have overall 10 different RCM models (10 times training) for hair structure and 10 different RCM models for epidermis with randomly shuffled dataset.

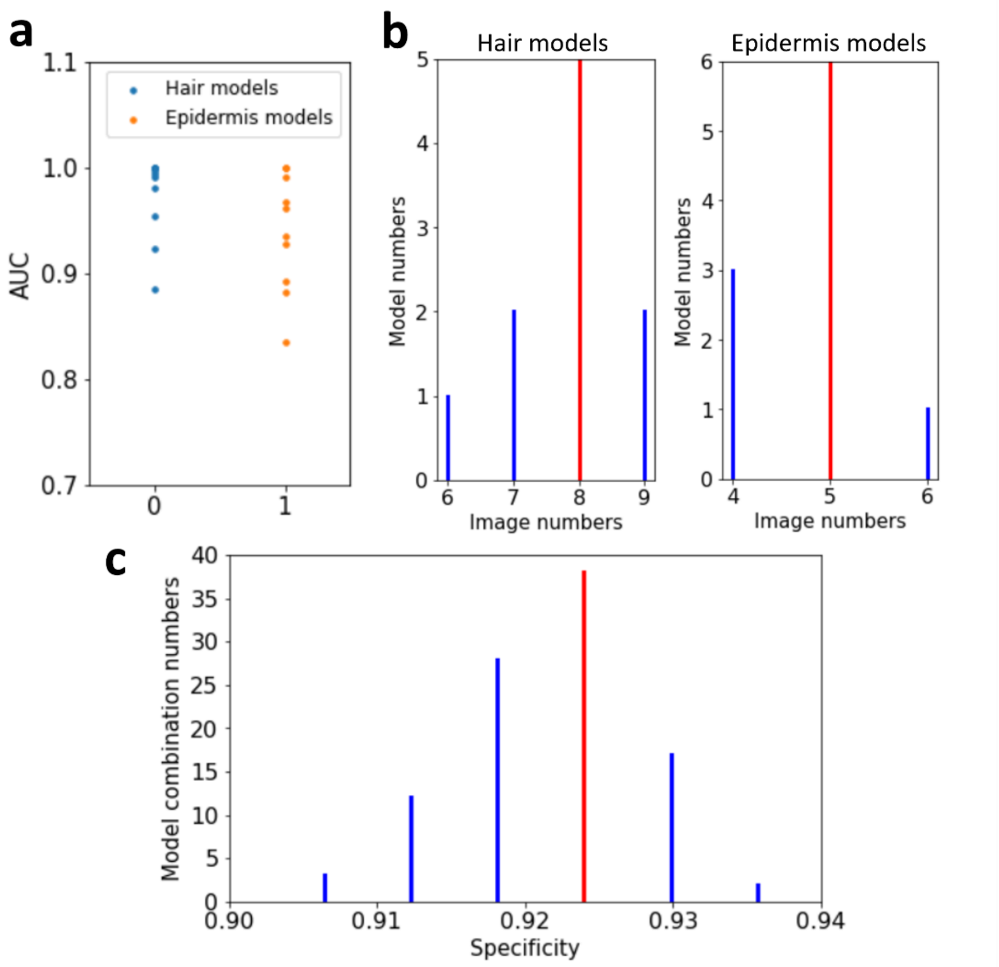

Fig. S3 10 times bootstrapping results for each model. (a) The AUC values on the test sets. Blue dots are the AUC values from hair models, orange dots are the AUC values from epidermis models. (b) The performance of the

models on the task group. The red lines are where our reported models belong to. (c) The histogram of the specificity. The red line is where our reported specificity belongs to.

Fig. S3 shows the results from all the 20 different models. In Fig. S3 (a), the AUC values on test sets are reported. The AUC values of hair models range from 0.88 to 1.00, and most values are close to 1.00. The AUC of epidermis models range from 0.83 to 1.00, and the distribution is relatively uniform between 0.88 to 1.00. All the models were also used on the task group to select hair and epidermis structure. Fig. S3 (b) shows their performance on the task group. X-axes represents the number of false positive (FP) images that were selected by the models, and Y-axes represents the number of models. For example, for the epidermis models, 3 models selected 4 FP images, 6 models selected 5 FP images and 1 model selected 6 FP images. The hair model reported in the paper selected 8 images, and the epidermis model reported selected 5 images. We had 10 hair models and 10 epidermis models, which generated 100 different combinations. Fig. S3 (c) shows the histogram of the specificity after applying these combinations. The red line illustrates the model of our reported specificity. From Fig. S3 we can see that random shuffling of the dataset did not make a significant impact on the overall performance, and our reported model represents an average performance of trained models.
